# Effects of visual pacing cue and motivational auditory content on running fatigue and biomechanics

**DOI:** 10.1371/journal.pone.0345554

**Published:** 2026-08-24

**Authors:** Megan Dreher, Alexis Terterov, Olivia Feistner, Laura Freiermuth, Polly Schaps, Haley Yeager, Janet Zhang-Lea

**Affiliations:** 1 Department of Human Physiology, Gonzaga University, Spokane, Washington, United States of America; 2 Department of Sport and Exercise Sciences, Durham University, Durham, England; 3 Department of Anthropology, Durham University, Durham, England; 4 Department of Human Physiology, University of Oregon, Eugene, Oregon, United States of America; NLA Hogskolen - Oslo, NORWAY

## Abstract

Rhythmic synchronization and auditory distraction are mechanisms commonly proposed to explain the role of music in delaying fatigue-related responses in endurance sports. However, the specific contribution of each mechanism remains unclear. We designed a cross-sectional study and investigated the effect of rhythmic synchronization and auditory distraction on running biomechanics. Fifteen adults (age = 20.9±1.3 years, weight = 71.2±12.1 kg, height = 174.7±11.0 cm) ran at a moderate intensity on a treadmill for up to ten minutes, or until reaching predetermined termination criteria. Participants finished three trials, starting with running without any stimulus as a baseline trial, and ran with a visual metronome that flashed at a rate that matched their self-selected running cadence in the visual stimulus trial (VST). In the visual-auditory stimulus trial (VAST), participants ran with the visual metronome (as described in VST) while listening to a non-rhythmic motivational speech. We recorded run duration, perceived exertion, center of pressure sway during standing before and after each trial, and measured trunk acceleration to obtain root-mean-square (RMS) of acceleration during each minute of the run. Compared to baseline, participants reduced perceived exertion by 0.87 and 0.85 rating during the VST and VAST, respectively, though these changes did not reach significance (p = 0.05). Stimulus affected the RMS of acceleration in anterior-posterior (p = 0.011), vertical (p = 0.008), and resultant directions (p = 0.006). Our linear mixed effect model suggested that compared to VST, VAST further lowered RMS of acceleration by 0.026g (anterior-posterior), 0.028g (vertical), and 0.036g (resultant). Our study provides an approach to investigate the components of music via music proxies, and our findings highlight the effect of combining rhythmic synchronization and motivational auditory distraction on fatigue-related biomechanics variables during submaximal running. Due to the use of musical proxy stimuli within a limited run duration, our results should be interpreted as preliminary and not directly generalizable to real-world music listening conditions.

## Introduction

Motivational music has been found to elicit positive emotional responses in runners, to improve run performance, and to extend run duration [1,2]. The effect of music in impeding running fatigue has been attributed to the tempo and the motivational lyrics of music [3]. Music tempo could generate an *synchronous effect*, which occurs when runners temporally regulate their running cadence to the tempo of music [3]. Synchronicity induced by the tempo of music is shown to lower oxygen consumption in runners, indicating a reduced muscle activation and improved preservation of metabolic energy [4,5]. Synchronous music has been found to reduce perceived exertion and to increase run duration by up to 15% [6–8]. Prior studies show that runners adjust their running cadence, which is usually between 130 and 200 steps per minute [9], in accordance with a music tempo within 2.5–10% of their natural cadence to increase their running speed [9–11]. However, this adaptation may be influenced by the instructions of researchers rather than a natural entrainment of running cadence to music tempo [11].

On the other hand, motivational content stemming from music lyrics may divert attention from feelings of fatigue while running [3,12,13], and therefore could extend run duration through *distraction effect*, as described in the attentional processing mechanism [3,12]. The distraction effect of music is harder to quantify since all components of music can be argued as a distraction to the runners from feeling fatigued, which includes motivational lyrics, tempo, melody, etc. Previous studies aimed to assess the distraction effect of music, but did not separately study the effect of music tempo and the effect of motivational lyrics on running fatigue and running biomechanics [14]. Therefore, it is unclear whether the previously reported effect of music in delaying fatigue is solely due to the synchronous effect of music tempo or it’s a combination of tempo and motivational lyrics of music.

To our knowledge, prior research investigating the effects of music on running fatigue has not distinguished between the *synchronous* and *distraction* effects of music, making it difficult to discern which mechanism has the greatest impact on delaying running fatigue. We isolated two components commonly attributed to music effects: rhythmic synchronization and motivational auditory content, using non-musical proxies. We aim to use this approach to mechanistically investigate each component of music, but we do not aim to replicate real-world music listening conditions in this study. We will measure run duration, perceived exertion, and fatigue-related biomechanics variables to assess the onset of running-induced fatigue [15–17]. To simulate the *synchronous effect*, we utilized a flashing visual metronome to provide the visual pacing cue, and matched the frequency of the metronome at the runners’ self-selected running cadence. We hypothesized that compared to running without a visual pacing cue, runners would prolong their run, experience less perceived exertion, reduce deviations in their root-mean-squares (RMS) of trunk acceleration, and reduce their standing Center of Pressure (CoP) sway when given the visual pacing cue. To simulate the *distraction effect*, we utilized a non-rhythmic auditory motivational speech that was unfamiliar to participants, and we hypothesized that compared to running with only the visual metronome, runners would further prolong their run, experience less perceived exertion, reduce RMS of trunk acceleration, and further reduce standing CoP sway after running when listening to the motivational speech.

## Methods

### Participants

We conducted a prior sample size estimation using G*Power [18] based on previously published effect size values. Since to our best knowledge, no study has reported the effect of *synchronous effect* or *distraction effect* on root-mean-squares (RMS) of trunk acceleration, or CoP, we used previous publications reporting the effect of music on oxygen consumption (Cohen’s f = 1.03) [5], electromyographic fatigue threshold (Cohen’s f = 1.94) and lower limb muscle maximal power output (Cohen’s f = 1.66) [19]. We set the power at 0.8, and the type I error rate at 5%, and obtained an estimated sample size of 4–8 participants. However, these estimates were based on large effect sizes reported in prior literature using physiological outcome variables. We expect our results to provide preliminary investigation.

Fifteen young adults (7 males, 8 females, age = 20.9 ± 1.3 years, body weight = 71.2 ± 12.1 kg, body height = 174.7 ± 11.0 cm) participated in this study. Participants were over 18 years old and self-reported to be engaged in a variety of moderate-intensity exercise regularly and have previously used a treadmill for moderate-intensity exercise in the past 6 months. All participants were free of cardiovascular, neurological, and musculoskeletal conditions. Additional exclusion criteria included untreated vision or hearing loss as well as a history of epilepsy or light sensitivity. This study was approved by the Gonzaga University Institutional Review Board, and the recruitment period started June 16, 2023, and ended on May 15, 2024. Written informed consents were obtained from all participants prior to their participation.

### Experimental protocol

All participants first completed an overground sprinting trial for us to obtain their maximum speed. Participants accelerated for 20 meters before running through two speed gates (DASHR, Lincoln, Nebraska) spaced 3 meters apart. We recorded the average velocity within the 3-meter distance between the two speed gates as the participant’s maximal, reported in m/s. Participants were given three times to sprint through the speed gates, we took the fastest speed among the three trials as the individual’s maximum speed. We used 55% of each participant’s maximum speed as their testing speed.

**Fig 1:**
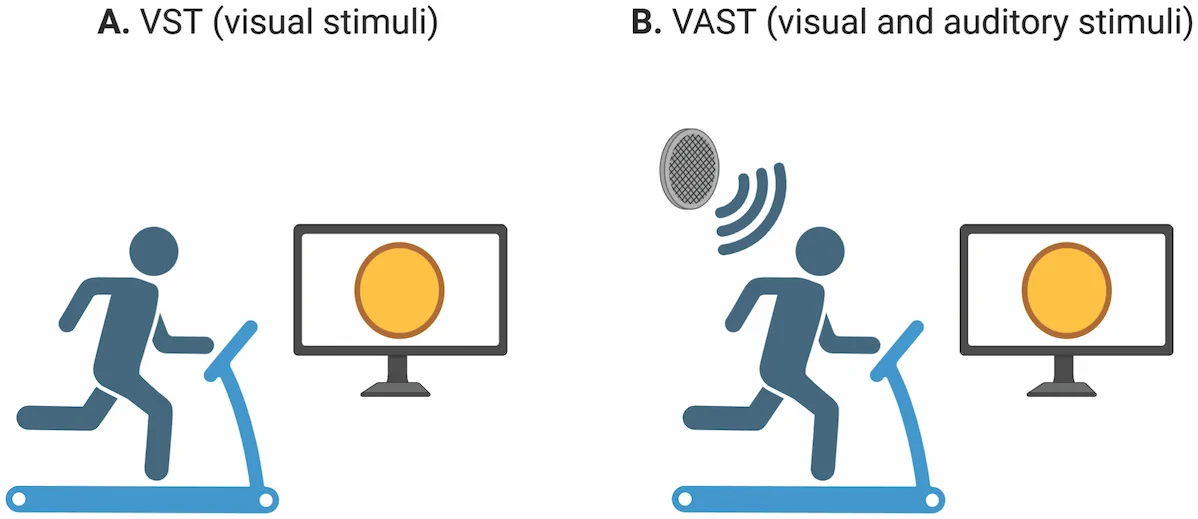
Overview of experimental setup for visual stimulus (VST) and visual-auditory stimulus trials (VAST). Participants completed all three treadmill running trials at 55% of their maximum speed, and ran with no feedback during baseline trial. In VST (A) and VAST (B), participants ran with a visual metronome shown as a flashing yellow circle on a screen positioned in front of the treadmill at eye level. Additional auditory stimulus was provided during VAST via a speaker positioned on the treadmill at the same spot for every trial (B).

Participants then completed baseline, visual stimulus (VST), and visual-auditory stimulus (VAST) treadmill (Woodway, Waukesha, Wisconsin) running trials at testing speed on separate days with at least five days between trials (Fig 1). All participants completed a structured, dynamic warm-up led by one of our experimenters prior to each run. When beginning each run, we hid the speed and running time from participants and increased the speed by 0.45 m/s every 3 seconds until the testing speed was reached. A Polar Heart Rate Chest Strap (Polar, Kempele, Finland) was fixed to a participant’s sternum monitoring heart rate (HR) continuously and maximum HR was defined as the sum of 220 minus a participant’s age [20]. Participants were asked to verbally rate themselves on the Borg Rating of Perceived Exertion Scale (RPE) as a measure of perceived fatigue [15]. HR and RPE were recorded every minute. Trials were terminated when (A) maximum HR was reached, (B) a score of 17 was reported on the RPE, (C) the treadmill safety clip was pulled, or (D) the trial lasted for ten minutes. Participants were blinded to the duration of each trial. Participants wore a triaxial accelerometer (±16g, Delsys, Natick, MA) on the back of their L1 vertebrae, secured by athletic tape, throughout the duration of each trial. The accelerometer measured trunk acceleration along the medio-lateral, vertical, and anterior-posterior axes at 148 Hz. Immediately before and after each running trial, Participants stood hip-width apart on a Kistler force plate (9287BA, Kistler Group, Novi, Michigan) for 10 seconds and we recorded CoP trajectory at 1000 Hz using BioWare (Kistler Group, Alberta, Canada).

All participants completed baseline trial first and completed VST and VAST in a randomized order. During baseline running, we used a smartphone (iPhone 12, Apple, Cupertino, CA) to record the first ten seconds of the participant running at test speed at a 60 Hz frame rate. This footage was used in conjunction with a mobile phone application (Metronome, Soundbrenner, Hong Kong, China) to calculate cadence as the total number of steps a participant took per minute. A visual metronome allowed us to provide rhythmic pacing without introducing auditory rhythmic cues that could confound the motivational auditory condition. We calibrated a custom-written visual metronome (MATLAB, R2022B, MathWorks, Natick, Massachusetts), to flash a yellow circle at the same frequency as the participant’s cadence obtained at baseline. During VST, the visual metronome was displayed on an eye-level screen in front of the treadmill, and all participants were instructed to couple their running steps with the flashes (Fig 1A). During VAST, participants were instructed to couple their running steps with the same visual metronome while listening to a 10-minute audio recording (Fig 1B), which did not include any melody or rhythmic stimulation [21]. Using a visual metronome in this study allowed the participants to use this visual cue to guide their cadence while being able to receive auditory motivational content through the recording that is non-rhythmic. We played the audio recording via a speaker placed on the treadmill’s operating console, and the recording was played at a predetermined volume. We determined the volume of the speaker during our pilot testing at varied treadmill speed to make sure that the speech can be heard clearly when the treadmill was running. We kept the volume the same for all trials and all participants.

The motivational speech we chose for this study was delivered by former American basketball coach, Jim Valvano, on March 4, 1993, shortly before he passed away due to cancer. We chose this speech for many reasons. First, the length of the speech is 10 minutes 16 seconds, which aligns with the maximal run duration of our study (10 minutes). This is important because it allows our participants to be able to follow the paragraphs of content delivered by the speaker to the maximal level. Second, the theme of the speech aligns closely with sports and exercise. The speaker, Jim Valvano, was a basketball coach, and the speech was delivered when receiving the Excellence in Sports Performance Yearly award. In the speech, he openly spoke about having terminal cancer and showcases his resilience in the face of mortality, and the theme “Don’t give up. Don’t ever give up” runs through the entire speech. Third, this speech does not have any music component (e.g., rhythm, drumbeats) in the background, reducing confounding factors that may complicate the findings of our study.

### Data analysis

We first verified the cadence each participant ran with during each stimulus trial with the flash rate set at the visual metronome. We used a custom written program to identify ten peaks in the vertical acceleration data for ten consecutive steps in the middle of the trial. The time intervals between each step were used to calculate the participant’s self-selected cadence.

We filtered CoP data and trunk acceleration using a 4^th^ order Butterworth low-pass filter with a cutoff frequency at 30 and 50 Hz, respectively. We then performed a principal component analysis on CoP data and fitted a 95% confidence interval ellipse to CoP trajectory during standing. We defined the major axis length of the confidence ellipse as the frontal sway, and the minor axis length as the sagittal sway. For the trunk acceleration data, we first adjusted for gravitational acceleration for each axis and segmented the continuous acceleration data into 1-minute intervals. For participants with a final segment shorter than 1-minute, we reserved the data points if there were more than 10 seconds of running in the segment. We used a previously reported method to calculate root-mean-squares (RMS) of the trunk acceleration data [17]. Specifically, for each segment of data, we calculated the resultant acceleration and calculated the RMS of acceleration in the medio-lateral (ML_RMS_), anterior-posterior (AP_RMS_), vertical (VT_RMS_), as well as a resultant (RES_RMS_) directions [17]. We then divided the RMS of acceleration along each axis with the resultant RMS of acceleration to get the ratio of acceleration (ML_ratio_, AP_ratio,_ VT_ratio_).

### Statistical analysis

The Shapiro-Wilk test was used to test for data normality. If the normality assumption was met, we used one-way repeated measures ANOVAs to verify if the participant’s cadence during VST and VAST match with rate of visual metronome flashes. We used one-way repeated measures ANOVAs to compare the time to fatigue across all trials. We constructed several linear mixed models to compare the effects of running time (abbreviated as RT: 1^st^, 2^nd^, 3^rd^, etc. minute of running), stimulus condition (abbreviated as STIM: baseline, VST, VAST), and the interaction effect between RT and STIM on RPE and acceleration-related variables (RMS and RMS ratio). We constructed linear mixed models to compare the effects of trial completion (pre vs. post) and STIM on standing sagittal and frontal sway of CoP. All statistical tests were performed using MATLAB, with level of significance set at 0.05. If post-hoc analysis is needed, we conducted all post-hoc analysis in G*Power [18].

## Results

One participant stopped their VST by accident, and their data were excluded from all further analysis. Two participants were unable to record CoP data post-trial. The accelerometer detached from four participants before they reached fatigue, and their data were excluded from analysis of time to fatigue, cadence, RPE, and all trunk acceleration variables. Among all 15 participants, 12 were included in analysis of CoP, and 10 were included in the analysis of RPE and acceleration-related variables. Participants ran at similar cadences (F (2,27) =0.027, p = 0.97) in baseline (171.2± 10.4 SPM), VST (171.6± 12.7 SPM), and VAST (171.3± 13.4 SPM), and with similar run duration (F (2,27) =0.038, p = 0.96, baseline: 350.26 ± 192.80 s, VST: 371.01±189.30 s, VAST: 369.60± 185.80 s). Participants that stopped prior to 10-minutes also presented similar run duration across the three trials (F (2,18) =0.15, p = 0.86, baseline: 241.21 ± 97.33 s, VST: 269.57 ± 117.17 s, VAST: 265.20 ± 96.90 s).

### Rate of perceived exertion when running with varied stimulus conditions

**Fig 2:**
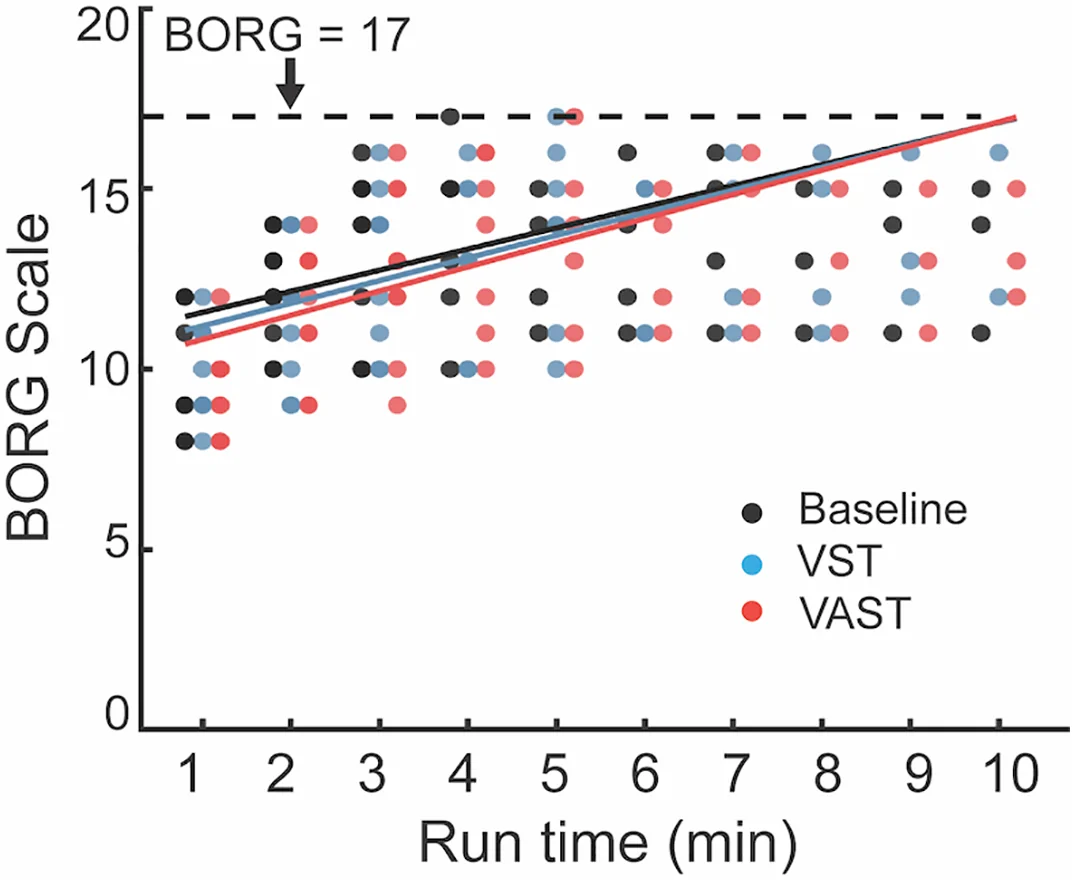
Effect of run time and stimulation on perceived fatigue, measured using the BORG scale. Each individual dot represents data from one participant. The three solid lines represents regression model generated based on linear mixed effect model for baseline (black), VST (blue), and VAST (red) trials. The dotted line represents BORG scale reaches 17, which is one of the criteria to stop the trial.

Run time had a significant effect on RPE (F = 91.13, p < 0.001), such that RPE increased by 0.585 for every minute increase in running time (p < 0.001), and STIM trended towards having an impact on RPE (F = 3.761, p = 0.05) but did not reach significance (Fig 2). Linear mixed models showed that compared to baseline, participants reported RPE values were 0.87 less during VST (p = 0.05) and 0.85 less during VAST (p = 0.05). RPEs between VST and VAST were not different (p = 0.97). We did not find an interaction effect between RT and STIM on RPE, F = 0.93, p = 0.34).

### Static CoP sway before and after running with varied stimulus conditions

**Fig 3:**
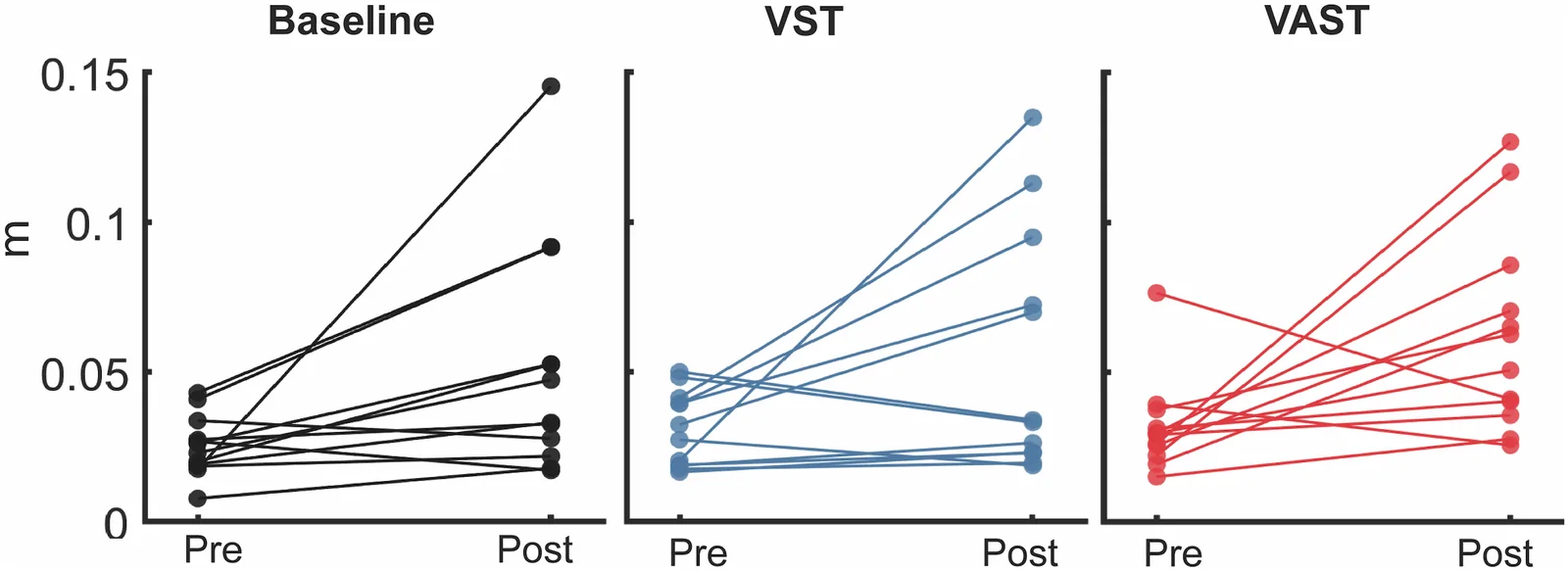
Static center of pressure sway increased after trial completion but did not vary between trials. Boxplots representing pre- and post-trial static CoP sway data for baseline (white), VST (grey), and VAST (black). All data is reported in the unit of meters.

When controlling for STIM and interaction effect, compared to pre-running, CoP_frontal_ increased by 0.026 m^2^ (p = 0.013) and CoP_sagittal_ increased by 0.017 m^2^ (p = 0.001) after completion of running trials (Table 1). When controlling for trial completion and interaction effect, STIM did not affect static CoP sway along the frontal (p = 0.56) or sagittal axis (p = 0.53) (Fig 3). We did not find any interaction effects between trial completion and stimulation for either CoP_frontal_ (p = 0.833) or CoP_sagittal_ (p = 0.416).

**Table 1:** Linear mixed model parameters for fixed effects of Trial Completion (pre-running vs. post-running) and Stimulus (baseline, VST, VAST) on static CoP sway along the frontal and sagittal axes.

CoP_frontal_ (m)	*Estimate (B)*	*SE*	*t*	*p*
Intercept	0.026	0.007	3.67	**< 0.001**
Trial Completion*	0.026	0.010	2.56	**0.013**
Stimulus^$^	0.003	0.005	0.59	0.558
Trial Completion x Stimulus	0.001	0.011	0.21	0.834
**CoP**_**sagittal**_ **(m)**	***Estimate (B)***	***SE***	***t***	***p***
Intercept	0.013	0.004	3.75	**< 0.001**
Trial Completion	0.017	0.005	3.40	**0.001**
Stimulus	0.002	0.003	0.63	0.533
Trial Completion x Stimulus	−0.003	0.005	−0.82	0.416

Reported with coefficient estimates (B), coefficient standard errors (SE), t values (*t*), and p values (*p)*.

* Continuous variable: pre-running as 0 vs. post-running as 1

$ Continuous variable: Baseline as 0 vs. VST as 1 vs. VAST as 2

### RMS of acceleration and ratio of acceleration when running with varied stimulus conditions

**Fig 4:**
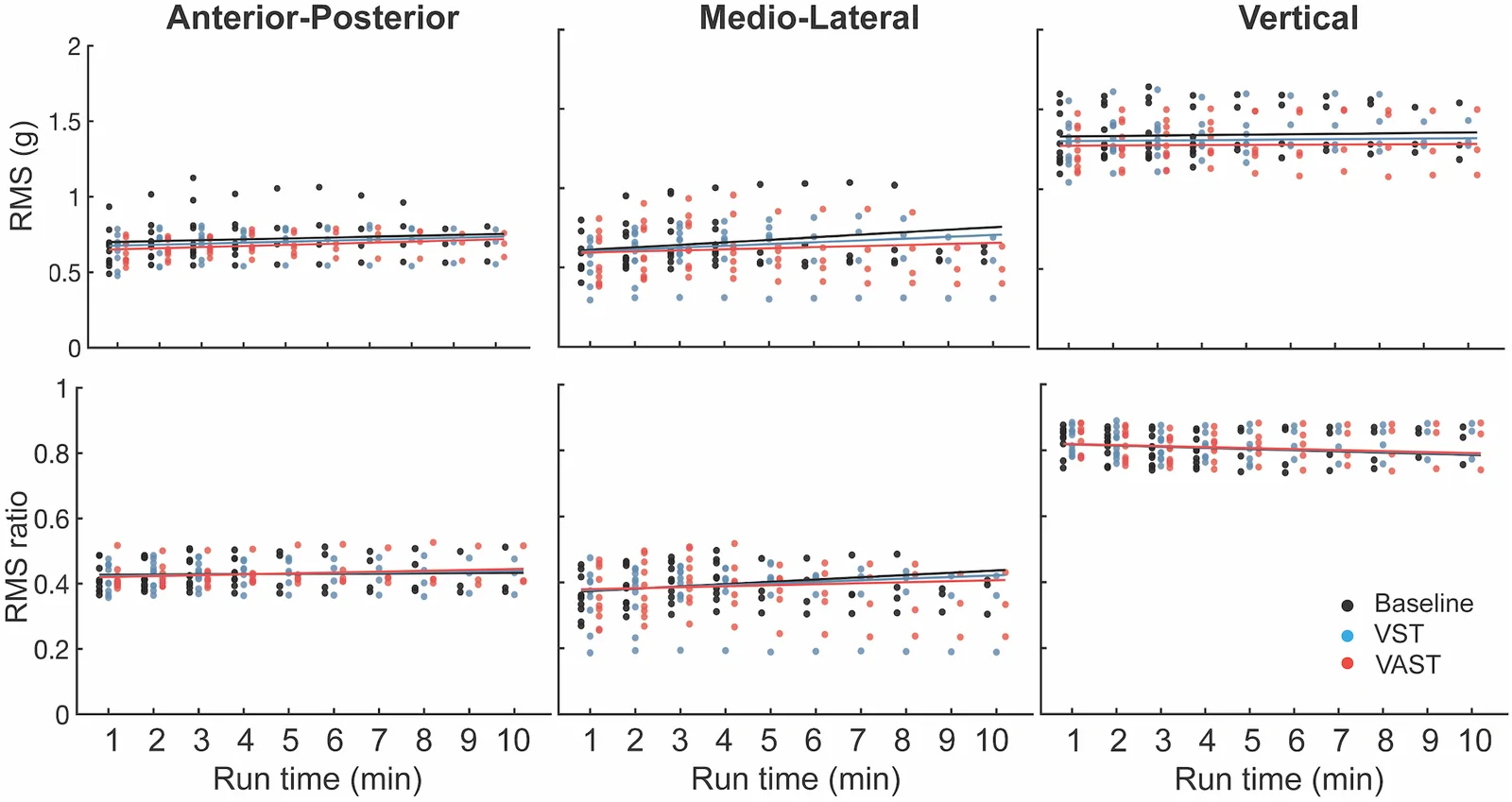
RMS of trunk acceleration along the medio-lateral, anterior-posterior, and vertical directions was lowest for VAST. RMS of trunk acceleration reported via boxplot for baseline (white), VST (grey), and VAST (black) for each minute of running time.

When controlling for STIM and interaction effect, running time affected the RMS of acceleration in the anterior-posterior (p = 0.037), medio-lateral (p < 0.001) and resultant directions (p = 0.001), but not in the vertical (p = 0.35) direction (Fig 4). Specifically, AP_RMS_ increased by 0.006 g, ML_RMS_ increased by 0.016 g, and RES_RMS_ increased by 0.011 g (Table 2) for every minute of running. When controlling for running time and interaction effect, STIM affected the RMS of acceleration in anterior-posterior (p = 0.011), vertical (p = 0.008), and resultant directions (p = 0.006), but not in the medio-lateral direction (p = 0.79, Fig 4). Based on the linear mixed effect model, adding one type of stimulation lowers AP_RMS_ by 0.026 g, VT_RMS_ by 0.028 g, and RES_RMS_ by 0.036 g (Table 2). We did not find any interaction effect between STIM and run time.

**Table 2:** Linear mixed model parameters for fixed effects of Running Time (min) and Stimulus (baseline, VST, VAST) on RMS of acceleration.

AP_RMS_ (G)	*Estimate (B)*	*SE*	*t*	*p*
Intercept	0.694	0.030	23.10	**< 0.001**
Run Time (Minutes)	0.006	0.003	2.10	**0.037**
Stimulus*	−0.026	0.010	−2.58	**0.011**
Run Time x Stimulus	<0.001	0.002	0.40	0.688
**ML**_**RMS**_ **(G)**	***Estimate (B)***	***SE***	***t***	***p***
Intercept	0.592	0.046	12.93	**< 0.001**
Run Time (Minutes)	0.016	0.003	4.56	**< 0.001**
Stimulus	−0.003	0.025	−0.26	0.794
Run Time x Stimulus	−0.004	0.024	−1.79	0.076
**VT**_**RMS**_ **(G)**	***Estimate (B)***	***SE***	***t***	***p***
Intercept	1.327	0.041	32.57	**< 0.001**
Run Time (Minutes)	0.003	0.003	0.94	0.347
Stimulus	−0.028	0.011	−2.69	**0.008**
Run Time x Stimulus	<−0.001	0.002	−0.36	0.722
**RES**_**RMS**_ **(G)**	***Estimate (B)***	***SE***	***t***	***p***
Intercept	1.617	0.051	31.86	**< 0.001**
Run Time (Minutes)	0.011	0.003	3.049	**0.003**
Stimulus	−0.036	0.013	−2.78	**0.006**
Run Time x Stimulus	−0.002	0.003	−0.72	0.475
**AP**_**ratio**_	***Estimate (B)***	***SE***	***t***	***p***
Intercept	0.426	0.011	38.60	**< 0.001**
Run Time (Minutes)	<0.001	0.001	0.58	0.560
Stimulus	−0.005	0.004	−1.10	0.274
Run Time x Stimulus	0.008	<0.001	1.22	0.225
**ML**_**ratio**_	***Estimate (B)***	***SE***	***t***	***p***
Intercept	0.366	0.023	16.23	**< 0.001**
Run Time (Minutes)	0.007	0.002	4.00	**< 0.001**
Stimulus	0.005	0.006	0.79	0.428
Run Time x Stimulus	− 0.002	0.001	−1.55	0.123
**VT**_**ratio**_	***Estimate (B)***	***SE***	***t***	***p***
Intercept	0.822	0.013	63.21	**< 0.001**
Run Time (Minutes)	−0.003	0.001	−3.32	**0.001**
Stimulus	<0.001	0.003	0.006	0.995
Run Time x Stimulus	<0.001	<0.001	0.32	0.749

Reported with coefficient estimates (B), coefficient standard errors (SE), t values (*t*), and p values (*p)*.

* Continuous variable: baseline as 0 vs. VST as 1 vs. VAST as 2

When controlling for STIM and interaction effect, running time affected the ratio of RMS of acceleration in medio-lateral (p < 0.001) and vertical (p = 0.001) directions, but not anterior-posterior (p = 0.756). Specifically, ML_ratio_ increased by 0.007 (p < 0.001) and VT_ratio_ decreased by 0.003 (p = 0.001) (Table 2) for every minute running. When controlling for running time and interaction effect, STIM did not affect the ratio of RMS of acceleration in the anterior-posterior (p = 0.304), medio-lateral (p = 0.266), or vertical (p = 0.789) directions. Running time did not interact with STIM on any of the directions (p = 0.225 for AP_ratio_, p = 0.123 for ML_ratio_, p = 0.749 for VT_ratio_).

## Discussion

Through measures of run duration, perceived exertion, and fatigue-related biomechanics variables, this study investigated the effects of both visual pacing cue and auditory non-rhythmic motivational content on fatigue-related variables. Participants ran on the treadmill at 55% of their maximal sprinting speed for 10 minutes, or until exhaustion, with no stimulus, with a visual metronome, and with a non-rhythmic motivational audio recording. We found that visual pacing cue was responsible for improvements in perceived exertion compared to baseline, while the combined effect of auditory motivational content and the visual pacing cue was responsible for lower RMS of trunk acceleration compared to baseline.

### Rhythmic synchronization from the visual metronome is responsible for reduced perceived exertion compared to baseline

A key methodological consideration of this study was the use of a visual metronome to simulate rhythmic synchronization. Prior research has predominantly employed auditory rhythmic stimuli due to the strong coupling between auditory perception and motor timing. In contrast, visual pacing cues may engage different perceptual and sensorimotor processes. Therefore, the effects observed in this study should be interpreted as responses to externally guided temporal synchronization rather than direct evidence of auditory rhythm or music tempo effects.

Despite the difference lying in between visual metronome and auditory rhythmic stimuli, our study showed very similar results as previous research studies using auditory rhythmic stimuli [4,8,22]. We partially accepted our first hypothesis, that participants’ RPE trended towards being greater at baseline compared to both VST and VAST, but we did not find any difference in RPE between VST and VAST. The frequency of the beat was matched with the runner’s self-selected cadence, which could amplify the *synchronous effect* [4,8,22]. Some prior studies have proposed that using an auditory metronome in running enabled runners to anticipate their next step and to maintain their optimal cadence when it would otherwise slow down due to fatigue [8,12].

We found similar RPE between VST and VAST (p = 0.97), and we therefore suggest that the *synchronous effect*induced via the visual metronome is more responsible for a lower RPE compared to the *distraction effect* induced via auditory motivational content. Such results are in conflict with some previous studies suggesting the distraction effect of music created by motivational content is also responsible for a lower RPE [1,23]. One possible explanation is that the previous studies allowed participants to self-select their preferred motivational music, while all our participants listened to the same motivational speech. Listening to the same speech allowed us to isolate and control the auditory content delivered to runners, but it is possible that not all participants were similarly engaged with the motivational speech due to personal preference. Our study found that run duration and CoP sway during standing did not differ across trials, indicating that neither the *synchronous effect* nor *distraction effect* improve this aspect of running performance. In our study, only seven participants reached our definition of fatigue when each trial stopped, which limits our interpretation of the effect of stimulation on run duration.

### Auditory distraction and rhythmic synchronization together reduced RMS of trunk acceleration compared to baseline

We partially accepted our second hypothesis. Our results suggested that VAST did not further increase run duration or lower RPE values compared to VST. However, we found that the type of stimulation had an impact on RMS of trunk acceleration in the anterior-posterior, vertical, and resultant directions. Specifically, when controlling for time and interaction effect, running with VAST further lowers RMS of trunk in the previously mentioned directions compared to baseline condition. An increase in the RMS of acceleration has previously been attributed to running fatigue, as it is suggested to result from the decrease in the knee flexion angle of a runner [24] during fatigued status. Thus, the lower RMS of acceleration observed in each of these directions in VAST compared to baseline suggests that the combined use of a visual metronome and motivational content reduced fatigue-related biomechanics variables.

Our further pairwise comparison suggested that RMS of trunk acceleration trended towards reduction when comparing VST to baseline, but such difference did not reach significance (p > 0.064 for all directions). This result suggests that combining motivational content with the visual metronome best reduced fatigue-related biomechanics variables, as opposed to findings that credit only the *synchronous effect* but not the *distraction effect* from motivational content [7,8]. Such discrepancy in our findings could be due to the fact that we did not try to create a realistic music-listening environment, but we aimed to investigate the mechanism of two components using music proxies. As a result, previous study had runners exposed to different music pieces in order to match the music beats to their cadence, while in our study, we controlled the motivational content that runners listened to by isolating rhythmic synchronization and motivational auditory content. Our findings suggested that *distraction effect* on its own has impact in lowering fatigue-related biomechanics variable. There have been studies reporting benefits of external focus or distraction to endurance sports, including a better running economy, an increased VO_2max_, which are all potentially contribute to a delayed fatigue in endurance running [25–27]. One proposed explanation is that when exposed to external distraction, runners shift their focus from internal behavior, such as stride length and step frequency, to external environment, such as breathing or external environment [25,28].

A key limitation of this study is that the stimuli used (visual metronome and non-rhythmic motivational speech) do not capture the multidimensional nature of real-world music, which includes melody, harmony, lyrics, familiarity, and personal preference. Therefore, the findings should be interpreted as mechanistic rather than directly generalizable to music listening during running. Our study quantified fatigue using one accelerometer attached on the trunk, which is considered to associate with running kinematic changes that occur during running fatigue. However, we did not directly measure running kinematics variables in this study. We limited the maximum running duration to 10 minutes, and we expected our participants to reach fatigue prior to 10 minutes. While majority of our participants stopped prior to the 10-minute time mark, there were three participants able to finish all 10 minutes of running. Consequently, the observed changes in trunk acceleration may reflect early or submaximal fatigue responses rather than fully fatigued states. This limitation may also explain the lack of significant differences observed in run duration and perceived exertion between conditions. Future studies should consider longer or individually calibrated protocols to ensure consistent induction of fatigue across participants and improve sensitivity to fatigue-related adaptations.

## Conclusions

Our study found that the *distraction effect* from the auditory motivational content showed additional impact on lowering fatigue-related biomechanics variables under submaximal running conditions compared to *synchronous effect* only condition. While our results suggest potential benefits of combining rhythmic synchronization with motivational auditory content, future studies using ecologically valid music stimuli are required before making specific recommendations for music use during running. Additionally, in situations when runners are not allowed to listen to music, or when music is not available, motivational talks could potentially affect fatigue-related biomechanics variables during submaximal running even if they are non-rhythmic and do not align with the runner’s cadence.

## Supporting information

S1 File Supplementary files. **S1.** Acceleration and RPE data. **S2.** CoP data. **S3.** Script: MATLAB script for statistical analysis.
(ZIP)
